# Removable and Fixed Bridgework*This is an extract from a letter by Dr. Frahm to the editor of *Dental Items of Interest*, which is printed in the November number. It is well worth studying.

**Published:** 1920-02

**Authors:** F. W. Frahm


### REMOVABLE AND FIXED BRIDGEWORK*

BY F. W. FRAHM, D.D.S.

What are the convictions of the profession regarding a properly constructed fixed bridge, and what are our professional convictions regarding removable bridgework of the present day? After considering all the merits and demerits of these two classes of bridges, shall we insist on the removable type to the exclusion of the other? Have we advanced far enough in removable bridgework to enable us to take a very definite stand? Whatever the answer may be, let us, for the time, consider the dictating patient. No matter what our attitude may be, the potent factor in the choice of a bridge remains, to a large extent, with the patient. A surgeon may have well-grounded convictions that are born of scientific knowledge and experience regarding the necessity for performing a certain surgical operation on his patient, in order that he may recover his health. However, the patient may choose to have the operation performed, or to take his chances on recovery without such an operation, and the surgeon will have to abide by that decision, regardless of his desire to prolong life. The case may be serious, or even grave, but the surgeon must stand idly by, violate his "professional conscience," and allow his patient to pass out of his sphere of activity. I think I can hear you say: "But, doctor,

*This is an extract from a letter by Dr. Frahm to the editor of *Dental Items of Interest*, which is printed in the November number. It is well worth studying.

the surgeon here is on different ground from that of the bridgeworker, in that he is not doing the patient an injury, and his position is somewhat passive, thus allowing nature to take her course.'' Let us see. The patient wants the physician to handle the case with therapeutic agents and not surgically. The physician may refuse in some states and not lay himself liable by such a refusal, while in others he would be subject to criminal neglect by his refusal to use the recognized agents which the materia medica offers. He has the same privileges we have, and must consider the wishes of the patient and his family. That is just what we do when we recommend one type of bridge, but yield to the patient's wishes against our judgment.

It simply resolves itself into this—we are not called merely to serve an ideal, right or otherwise, but to serve humanity to the best of our ability in so far as the said humanity will co-operate, or allow us to do for them the things that should be done. This may not seem ideal; however, it is an exceedingly practical procedure in this practical world. It may not be the ultra in ethics for the bridgeworker, but it is the only reasonable method of practice so long as our removable bridgework is in the transition stage, and we are not able to offer the patient real value for his money and a correction of the condition.

As for myself, I do not think the profession has developed at this time a removable bridge system of sufficient merit to be recognized as standard, or that can hope to fill the needs of all conditions in every case. Neither do I feel justified in condemning all fixed bridges, insisting that they shall be replaced with one of our present types of removable bridges. I shall gladly welcome something that will serve our patients a reasonable length of time without causing an injury, if it can be decisively proven that all fixed work militates against our ideals.

Most men, even including the dentists, are very similar to every other class of men. Likewise, it seems to be just as easy for the younger man to follow the line of least resistance as it is for the older man. The young graduate may have been taught the recognized and accepted methods of practice. He may have been shown the shortcomings of the methods of practice used only a few years ago; the shell crown and all its appendages may have been removed from his surroundings, and the odoriferous and septic bridge only known to him as a museum specimen—an evidence of an ancient form of practice. He may have been safeguarded against pernicious methods of practice by up-to-date, conscientious teachers, and he may have determined to do nothing that would contribute to a patient's ill health. But, lo! He has a rude awakening when he enters the practice of dentistry for himself. He finds the methods he was taught are idealistic, rational and satisfactory, but are not practiced by a very large percentage of his fellows. What shall this young man do? Well, he usually does what a good many other young men have done; he takes the line of least resistance—purchases some type of crown method that may be highly indorsed by a supply dealer, and he says: "If these other men, of good standing, can do it and get away with it, why should I not do it?" It may seem a small matter at the time, but *this young man has lost the vision* that the college has helped him to acquire. Having lost the vision, his mind and heart are soon fixed upon other things, the having of which is not wrong in itself. He wants a Pierce-Arrow or a similar means of locomotion. He wants only five- or six-hour days in an office that is luxurious, and equipped in the later styles. He seems to forget that the real things in dentistry can not be hung upon walls, nor placed around a room in a pleasing manner. Nor can he set them aside, because the dentist across the street, who may belong to the National and District Society, does things the other way.

Last week a young, able graduate of this year's class related this experience to me: "I have just returned from ———, where I took care of a dentist's office while he was on a trip east to take a special course in ————. A patient of this man came in to have a shell crown placed on a central which could have been restored better with a filling. I refused to do that kind of work and explained why it was refused. The patient left the office, went to another man up the street and had a shell crown made. You see, doctor, I lost $15 because I followed your teaching."

Such cases need to be multiplied only a time or two in the lives of men who are just entering practice, when some of the young men will fall and do as the other men in their town are doing. Can we blame them? Their loved ones need the funds that would come from such practice. They may have starved themselves to gain the coveted goal; now, why should they continue and fight for something the other men of the town will not practice? Why should we blame the colleges for the action of such men, or for the status of present-day crown and bridgework, or for the work done by some of the younger men of the profession? As a whole, the colleges are teaching and practicing far in advance of many of the old practitioners. Nevertheless, the colleges will continue to hold high ideals of practice, condemn indifferent methods, and work to establish rational methods.

Should our colleges refuse to teach a usual type or class of crown and bridgework, and present to the students some unproven thing that has not yet shed its swaddling clothes? Or something that has to be nursed and petted to keep it in presentable shape for a proving, and saying nothing about its working efficiency? I would question such a step, for we must remember that fixed bridgework is not entirely wrong in principle, but is largely wrong in practice and application by men who are indifferent to their patients' needs and well-being. If the construction engineer used as little judgment in building a railroad bridge as some dentists use in making and placing their bridges, the mortality rate among the traveling public would be greatly increased.

Should colleges refuse to teach fixed bridgework and try to teach all the "isms" and "cisms" now found in the field of removable work? After considering everything to date, I certainly would say *"No,"* and that with considerable emphasis. However, this is not the time nor place for me to discuss the kind and type of fixed bridge that I would use, or the cases where such a form of bridge is indicated. The writer would very gladly take up the discussion of rational bridgework with the sincere hope that order and something of value might be established by such a discussion in our journals. Honest and rational discussions can aid materially to bring order out of the chaos that now prevails.

It should not require a vivid imagination to picture a case or a condition where only a fixed bridge could be used and a removable bridge would be out of the question. Most of us have seen many such cases. It would be wrong for us to work up such a case in a manner wholly uncalled for, in order that a pet theory may be carried out. Likewise, it would be an injustice to the students if our colleges refused to teach them *safe and sane fixed bridgework* in conjunction with removable and semi-removable bridgework. I would go one step further and include in the student's required work a piece of the movable-removable bridgework, because I feel that the student should have such a training to enable him to meet general and special requirements. A thorough analysis of the work should be given and clinical cases shown that would present indications for each type of bridge, to enable the student to make a rational application of the principles involved.

Too often the student is trained to become a master mechanician in the art of bridgework, but is largely ignorant of the principles and science involved in the subject. His technical work on the bridge is everything that we might desire, but there is a neglect to consider pathological and anatomical conditions that would directly or indirectly militate against his best technical efforts. Such neglect will cause *any type of bridge to fail.* In other words, we should not always look for the reasons of the failure in the type of bridge, *but in the unfortunate selection for the case.*

I can not agree that the elimination of all strain from the abutments follows the use of removable work. That is to say, that removable bridges can be so constructed that the saddle will take all the strain and the natural teeth on one or both ends of the span will not be subjected to any strain. Such a premise is a delusion, and any one who would maintain such a position is deceiving himself, or is ignorant of the principles involved.

You say the removable bridge does not interfere in any manner with the treatment of the diseased abutments. Granted, in part; but why should we have diseased abutments in removable work? And, if the abutments were healthy when our removable bridge was made, why have they, in supporting such a bridge, become diseased and need treatment? If these abutments became diseased with the wearing of a removable bridge, why not acknowledge the facts, extract the teeth, and charge the failure where it belongs? Such diseased teeth will never again become healthy and strong members of the arch as long as they are called upon to *take care of the added strain imposed upon them by the removable bridge.* Allow me to assert, without fear of successful contradiction, *that every now-known type and form of bridgework as practiced to-day, places an unnatural strain on and overloads every tooth that acts either as a direct or indirect retainer. Ultimate disaster awaits all bridges, fixed or otherwise,* for we have not yet found a means of supplying lost dental masticating units, without placing strain upon one or more of the remaining units of the arch. Some one may perfect an implantation technic to which our human system will not object, and solve our problem of supplying the lost units. I would gladly hail such a day.

Can I refute the charges made against fixed bridgework? Let me say that I do not believe the charges have been sustained against the *science involved;* neither have the underlying principles suffered to any extent. The charges now lodged apply only to the *abuse* of the science and principles of fixed bridgework. I speak confidently and after deliberation when I say that fixed bridgework will arise and emerge from the present refining fires with a better technic and application of principles than would have been probable in any other way. These refining fires will burn away all that is undesirable and leave with us something standard and worth while.

Why should the college professor act as the buffer, or pull the chestnuts out of the fire, while the large majority of men are indifferent? I believe he is doing the best he can under the conditions prevailing at this time. Personally, I am open to receive anything that will prove of value and will bear clinical investigation. I am of the opinion that there are clinical cases that can only be cared for with a fixed bridge, and others that must be restored with a "semi-removable," removable, or even a "movable removable" bridge. Also, that there is no one type of cure-all in our bridge armamentarium.

